# The effect of diet on primary dysmenorrhea in university students: A randomized controlled clinical trial

**DOI:** 10.12669/pjms.346.16477

**Published:** 2018

**Authors:** Yasemin Aydin Kartal, Elvan Yilmaz Akyuz

**Affiliations:** 1 *Dr. Yasemin Aydin Kartal, Assistant Professor Department of Midwifery, Faculty of Health Sciences, University of Health Sciences, Istanbul, Turkey*; 2 *Dr. Elvan Yilmaz Akyuz Assistant Professor Department of Nutrition and Dietetics, Faculty of Health Sciences, University of Health Sciences, Istanbul, Turkey*

**Keywords:** Diet, Primary Dysmenorrhea, University Students

## Abstract

**Objective:**

To evaluate the effect of diet therapy on primary dysmenorrhea in female university students.

**Methods:**

A randomized controlled pre and post-test design was used to verify the effects of diet therapy on primary dysmenorrhea. The study was conducted on 67 female students who were suffering from primary dysmenorrhea. The participants were divided into diet and control groups. The intensity of dysmenorrhea was determined using Visual Analogue Scale and a modified questionnaire assessing several symptoms of dysmenorrhea. The intervention group received the diet therapy for three months. The assessments were made before intervention and three months later.

**Results:**

Before the intervention, the mean intensity of dysmenorrhea was found to be 7.14 ± 1.3, 7.09 ± 1.4, in diet groups and control groups, respectively, but the difference was not statistically significant. After the diet therapy, a significant difference was found among the two groups regarding the mean intensity of dysmenorrhea after three months and the average score of pain score of diet group was significantly lower (Diet group:5.15±1.15, Control group: 6.74±1.97).

**Conclusion:**

Diet therapy was found to be effective in reducing pain in female university students with primary dysmenorrhea complaints.

## INTRODUCTION

Dysmenorrhea is defined as painful menstruation and clinically characterized by varying degrees of pessary pelvic pain during menstruation. Pain is often spread to the waist, leg, inguinal region and rarely to the perineal and vesical regions. Dysmenorrhea is described as a gynecological problem that causes loss of work in addition to those that affect life negatively and bring emotional distress to the rest of the daily life activities.^1^,^2^ Dysmenorrhea can be accompanied by symptoms such as headache, dizziness, back pain, diarrhea, vomiting and fatigue.^3^,^4^ It has been reported that more than 90% of adolescent girls in the world and more than 50% of menstruating women complain of dysmenorrhea.^5^ When the situation in our country is examined, it is noteworthy that the frequency of dysmenorrhea ranges between 34% and 89.6%.^6^-^9^

Although it is widespread among young girls and affects daily life seriously, research shows that families and young people do not get adequate counseling and medical care for dysmenorrhea because they see it as a normal event in a traditional way. Because dysmenorrhea is perceived as a normal event, it is known that women are trying to relieve their pain with non-pharmacological methods rather than receiving treatment services. Non-pharmacological methods include lifestyle and diet changes, herbal medicines, and exercise. In diet therapy, balanced nutrition, low-fat diet, some herbal teas, reducing salt intake in the diet, fish oil, magnesium, zinc, Vitamins B and E and protein intake are effective approaches to reduce pain.^10^-^12^

Dysmenorrhea affects the lives of individuals in a negative way and can lead to decreased work productivity and work quality, increased accidents and absenteeism in school.^13^ For this reason, it is very important for young people to know the frequency of dysmenorrhea and the factors that affect them, their reactions to dysmenorrhea, the practices they are trying to cope with, and the problems related to dysmenorrhea. In the literature, suppression or exacerbation of symptoms of primary dysmenorrhea has been associated with some nutrients. There is a need for research that will help to improve nutrition protocols in order to investigate the relationship between primary dysmenorrhea and nutrition and thereby reduce the incidence of dysmenorrhea and alleviate symptom severity. Therefore, this study was conducted to determine the efficacy of the diet as a complementary treatment method in university students with primary dysmenorrhea.

## METHODS

This randomized controlled study was conducted after the ethics committee approval (No: 46418926-050.03.04) by Health Sciences University Hamidiye Clinical Research and informed written consent of the patients were received.

The population of the research consisted of first grade female students who were studying at School of Health Sciences and had dysmenorrhea complaints. Those who have pain in the abdomen, inguen, or waist region one day before the menstrual period and / or on the first day of menstruation were considered as “with dysmenorrhea”. The existence of dysmenorrhea that started and continued with the emergence of the first menstruation was defined as “primary dysmenorrhea”.^14^ Female students were followed for two cycles and assessed with VAS, and those who had primary dysmenorrhea complaints formed the population of the study (N:113).

The sample size was calculated before starting the study. In terms of outcome measures to be used, the type-I error was found to be the most significant in each sample group with α = 0.05 and 95% power value (1-β = 0.95) to find a significant difference between measurements both before and after treatment compared with the values of the visual analogue scale and it was found that there should be at least 28 individuals.^15^ It was planned to take 35 female students in each group considering the data losses to be experienced during the study period. A total of 70 students who agreed to participate in the study and were meeting the selection criteria were included in the study. Students were divided into two groups by closed envelope method which is one of the randomization methods. Which group the students will belong to was determined by randomly drawing the envelopes, with unknown content and already prepared by writing the names of the groups, by the students.

Diet therapy was applied to the first group (n=35) and the second group was assigned as the control group (n=35). Three students from the diet group left the study and a total of 67 students formed the sample of the study. The severity of primary dysmenorrhea was determined by using visual analogue scale and a questionnaire assessing the symptoms of dysmenorrhea. The case group applied the diet for three months (during three menstrual cycles). The assessments were made in two stages (before intervention and 3^rd^ month after).

Female students who were between 18 and 35 years of age, studying at the Faculty of Health Sciences of the public university, had BMI ≤18,5-≥29,9 kg/m^2^ and with primary dysmenorrhea complaint were included in the study. The exclusion criteria of the study were secondary dysmenorrhea, presence of diabetes, mental and physical disorder, endocrine drug use, endometriosis, pregnancy or lactacion, smoking and alcohol use, thyroid and heart disease, intrauterine device (IUD), policystic ovary syndrome, oral contraceptives and antidepressant use.

### Collection of the Data

Student Information Form and VAS were administered to the female students who formed the diet and control groups before the application (First Evulation). Diet therapy was administered to the students during three cycles (average three months). VAS was readministered to all groups three months after diet therapy was completed (Last Evulation).

The research data were collected by using the “Student Information Form” which includes the demographic information of the research participants and the “VAS” which was developed by the researchers and used to determine the intensity of the dysmenorrhea.

### Visual Analog Scale (VAS)

Students were asked to mark a suitable point on a horizontal line with a length of 10 cm in order to determine the severity of dysmenorrhea.

From the point of view of the current situation; 0: no pain at all, 10: maximum, so much pain to endure. Pain scale 1-4 is classified as mild pain, 5-6 as moderate pain, and 7-10 as severe pain. The point marked on the line was measured with a ruler and the severity of the pain felt by the students during menstruation was recorded in cm. Students rated the severest pain level during the day of menstruation on VAS after diet therapy.

### Diet Therapy

Foods consumed frequently by students were assessed and a diet compatible with primary dysmenorrhea was prepared by the expert dietician for the students. The students were given a diet compatible with primary dysmenorrhea and the diet was used during three menstrual cycles. In the diet content; adequate fluid intake and fibrous food consumption have been focused. Individuals had a diet containing 55% carbohydrates, <30% fat and 15-20% protein. The diet was rich in complex carbohydrates and fiber. Individuals have consumed fish for 1-2 times a week, > 1000 mg calcium and 8-10 nuts or 1-2 walnuts almonds per day. Also <300 mg caffeine restriction was made. The intake of spicy, acidic and carbonated foods was limited. The process of practicing the diet treatment of the students was followed by telephone interviews twice a week by the researcher.

## RESULTS

The mean age of the participants was 19.28 ± 1.31, the mean BMI was 20.99 ± 2.67 and the mean age of menarche was 13.31 ± 1.26. It was seen that 89.2% of the students had a menstrual cycle regimen between 21-35 days and 87.4% had a menstruation period changed between 2/7 days. When evaluated according to VAS, 25.8% of the students who complained of dysmenorrhea stated that they had moderate severity, 64.2% had severe dysmenorrhea, but more than half (58.6%) of the students did not receive medical help.

Details of the disorders associated with primary dysmenorrhea among the students are given in Table-I while the type of food consumed before intervention are shown in Table-II.

**Table-I:** Menstrual disorders associated with primary dysmenorrhea.

1. Back Pain	70.1%
2. Irritability	64.2%
3 Weakness	59.7%
4. Fatigue	49.3%
5. Ventricosity	47.8%
6. Depressive thoughts	41.8%
7. Pain in legs	37.3%
8. Breast Tenderness	35.8%
9. Frequent urination	32.8%
10. Nausea & Vomiting	22.4%
11. Concentration impairment	14.9%

**Table-II:** Type of food consumed by students before intervention.

1. Cereal group foods daily	44.8%
2. Egg, meat and similar foods	7.4%
3. Fish 1-3 times a week	21%
4. Fat and sugar-rich foods daily	17.9%
5. Vegetables 1-3 times a week	35.8%
6. Fruits 1-3 times a week	37.3%
7. A cup of coffee daily	37.3%

Prior to the intervention, the mean severity of primary dysmenorrhea was 7.14 ± 1.3, 7.09 ± 1.4 in the diet and control groups, respectively, and there was no statistically significant difference between the groups (Table-IV, p> 0.05). It was determined that there was a statistically significant difference between the two groups according to the severity of dysmenorrhea after three months of diet therapy and that the mean score of pain of the diet group was significantly lower.

**Table-III:** Methods used to cope with primary dysmenorrhea.

1. Rest	74.6%
2. Hot application	56.7%
3. Analgesics	41.4%
4. Herbal products	28.4%
Green Tea	28.4%
Ginger	16.9%
Chamomile tea	14.9%
Sage tea	7.9%
Fennel tea	6.8%
5. Massage	25.4%
6. Physical exercise	20.8%

**Table-IV:** VAS Evaluation Form Scores of Groups.

		Intervention Group (n:32)	Control Group (n:35)	

		Mean±SD	Mean±SD	Test*
VAS Total score	First Evaluation	7.14±1.3	7.09±1.4	* z:-,141
				p: 0,888
	Last Evaluation	5.15±1.15	6.74±1.97	*z: -1,23
				p: 0,000
	z/p	**z:-4,348	**z:-1,305	
		p:,000	p:,192	

* Mann Whitney U test,

** Wilcoxon Signed Ranks.

## DISCUSSION

This study was conducted to observe the effect of diet regulation on primary dysmenorrhea. In the study, the prevalence of dysmenorrhea was determined as 26.5% (N=426). It was reported in different countries that dysmenorrhea prevalence was between the ranges of 20% and 90%.^16^,^17^ When the situation related to dysmenorrhea prevalence in Turkey was examined, the prevalence between 34% and 89.6% was observed.^6^-^9^ Study results support the literature, and it is thought that the results of different researches vary due to regional and cultural differences.

Literature shows dysmenorrhea is not an inherited disorder, but it is closely related to family history. This is explained by the fact that dysmenorrhea is a learned behavior or psychological experience. In the study of Çıtak and Terzioğlu, it was stated that more than half (57.9%) of the students with dysmenorrhea history were experiencing dysmenorrhea and in the study of Erenel and Sentürk, it was stated that in two thirds of the students’ relatives were experiencing dysmenorrhea.^13^,^18^ Similarly, more than half (55.2%) of the students with dysmenorrhea in our study stated that they have first degree relatives such as mother or sister who had dysmenorrhea history.

Dysmenorrhea was reported to be localized in the lower abdomen region, spread to the waist, back and legs, and associated with vomiting, irritability, ventricosity, breast tenderness, headache and muscle cramps.^9^,^19^ In our study, similar to the literature, it was determined that the most common problems in patients with primary dysmenorrhea were low back pain (70.1%), irritability (64.2%), weakness (59.7%), fatigue (49.3%) and ventricosity (47.8%). These problems affect young girls’ lives, their daily activities and their productivity significantly. When these problems are considered, it is necessary to handle the primary dysmenorrhea which affects the students at a significant level from a multidisciplinary point of view by the healthcare professionals.

In our study, it was determined that 64.2% of the students who had dysmenorrhea had severe dysmenorrhea but that more than half (58.6%) of the students did not receive medical help. First of all, it is important for health professionals working in primary health care to be sensitive to this issue and to move students away from perceiving this situation as a situation that should be endured.

In the study, it was determined that methods of coping with primary dysmenorrhea were resting (74.6%), hot application (56.7%), analgesics (41.4%), herbal methods (28.4%), massage (25.4%) and physical exercise (20.8%). In Turan and Ceylan’s study, it was determined that 42.1% of students with primary dysmenorrhea were using analgesics, 26.1% were taking warm shower, 19.9% were using hot application and in Ersun and Zaybak’s study, it was determined that 76.7% of the students were using analgesics and 23.3% were changing positions and using hot application to reduce their pain.^20^,^21^ The diversity of methods used to cope with primary dysmenorrhea in research suggests that women in different countries / regions may be taking such a decision due to pain perception severity, reaction to pain, and cultural differences.

In the literature, there are many studies which show that there is a significant relationship between dysmenorrhea and nutrition type.^22^,^23^ In the study, it was determined that students frequently consumed food which are predisposing in primary dysmenorrhea (coffee, fat and sugar-rich foods). On the other hand, it was observed that they were consuming vegetable and fruit rich in fiber (vegetable, fruit) and quality protein sources (egg, fish, meat etc.) less. This situation shows the necessity of developing a nutrition protocol for students with complaints of primary dysmenorrhea. As a matter of fact, Barnard (2000) found that the severity of primary dysmenorrhea decreased significantly in women who were on a low fat diet.^10^ In the Cochrane systematic review, it was stated that B1 and B6 vitamins and fish oil (omega-3 fatty acids) are more effective than placebo in reducing pain severity in primary dysmenorrhea.^12^ In this study, the severity of dysmenorrhea in the diet and control groups was 7.14 ± 1.3, 7.09 ± 1.4 respectively after the diet therapy the severity of dysmenorrhea in the diet group was significantly lower than the control group (Control group: 6.74 ± 1.97, Diet group: 5.15 ± 1.15). The results of our study support the literature and it is seen that diet regimen is an effective non-pharmacologic treatment method in young girls to alleviate the severity of primary dysmenorrhea symptom.

### Limitations of the study

The follow-up period of the study was three months after the diet therapy. The study was only conducted in a public university.

## CONCLUSION

Our study showed that the frequency of pain severity in dysmenorrhea was significantly lower in the group treated with diet. It is suggested that healthy, lifestyle behaviors should be promoted in young girls and repeat such studies with a longer follow-up.

## RECOMMENDATIONS

The prevalence of primary dysmenorrhea is an important public health problem affecting quality of life, and it is quite common among young girls. The main goal in approaching female students with primary dysmenorrhea is to provide adequate relief in their pain.

### Authors Contribution

**YAK:** Conceived, designed and did statistical analysis, manuscript writing, editing of manuscript, final approval of the version to be published.

**EYA:** Did data collection, manuscript writing and diet therapy, final approval of the version to be published.
